# Socioeconomic Inequalities in the Retail Food Environment around Schools in a Southern European Context

**DOI:** 10.3390/nu11071511

**Published:** 2019-07-03

**Authors:** Julia Díez, Alba Cebrecos, Alba Rapela, Luisa N. Borrell, Usama Bilal, Manuel Franco

**Affiliations:** 1 Public Health and Epidemiology Research Group, School of Medicine, Universidad de Alcalá, Alcalá de Henares, 28801 Madrid, Spain; 2 Department of Surgery, Medical and Social Science, School of Medicine, Universidad de Alcalá, 28801 Madrid, Spain; 3 Department of Epidemiology and Biostatistics, Graduate School of Public Health and Health Policy, The City University of New York, New York, NY 10027, USA; 4 Urban Health Collaborative, Drexel Dornsife School of Public Health, Philadelphia, PA 19104, USA; 5 Department of Epidemiology and Biostatistics, Drexel Dornsife School of Public Health, Philadelphia, PA 19104, USA; 6 Department of Epidemiology, Johns Hopkins Bloomberg School of Public Health, Baltimore, MD 21205, USA

**Keywords:** children, adolescents, schools, food environment, health inequalities, socioeconomic status, spatial exposure, Spain

## Abstract

Across Europe, excess body weight rates are particularly high among children and adolescents living in Southern European contexts. In Spain, current food policies appeal to voluntary self-regulation of the food industry and parents’ responsibility. However, there is no research (within Spain) assessing the food environment surrounding schools. We examined the association between neighborhood-level socioeconomic status (NSES) and the spatial access to an unhealthy food environment around schools using both counts and distance measures, across the city of Madrid. We conducted a cross-sectional study citywide (*n* = 2443 census tracts). In 2017, we identified all schools (*n* = 1321) and all food retailers offering unhealthy food and beverages surrounding them (*n* = 6530) using publicly available data. We examined both the counts of retailers (within 400 m) and the distance (in meters) from the schools to the closest retailer. We used multilevel regressions to model the association of neighborhood-level socioeconomic status (NSES) with both measures, adjusting both models for population density. Almost all schools (95%) were surrounded by unhealthy retailers within 400 m (median = 17 retailers; interquartile range = 8–34). After adjusting for population density, NSES remained inversely associated with unhealthy food availability. Schools located in low-NSES areas (two lowest quintiles) showed, on average, 29% (IRR (Incidence Rate Ratio) = 1.29; 95% CI (Confidence Interval) = 1.12, 1.50) and 62% (IRR = 1.62; 95% CI = 1.35, 1.95) more counts of unhealthy retailers compared with schools in middle-NSES areas (ref.). Schools in high-NSES areas were farther from unhealthy food sources than those schools located in middle-NSES areas (β = 0.35; 95% CI = 0.14, 0.47). Regulating the school food environment (within and beyond school boundaries) may be a promising direction to prevent and reduce childhood obesity.

## 1. Introduction

Childhood obesity is one of the most important public health challenges globally [1]. Globally, about 213 million children and adolescents (aged 5 to 19 years) are overweight, with 124 million being obese [2]. Across Europe, rates of excess body weight are particularly high among children and adolescents living in Southern European countries [3,4]. In Spain, about 27% of children (aged 2 to 14 years) and 19% of youth (aged 15 to 17 years) were overweight or obese in 2017, respectively [5]. Being obese in childhood increases the risk of illnesses in adulthood (e.g., cardiovascular diseases) and premature mortality [6,7]. Obesity may be shaped by the development of healthy eating behaviors during childhood and adolescence [8]. Eating behaviors are important because they could help prevent or promote weight gain.

In general, eating behaviors are shaped by different physical, sociocultural, economic, and political factors, such as the food environment [9,10,11]. The food environment defines the foods available and accessible to children and adolescents. Furthermore, the influence of physical environmental factors (e.g., healthy food availability) may be shaped by social environmental factors (e.g., socioeconomic status) [10,11,12]. Children and adolescents spend a significant amount of time at schools, and thus, the school food environment has been considered a key arena for obesity prevention [13,14]. Yet, for most of the school-based intervention trials on childhood obesity prevention and control, results are mixed and modest [15,16,17]. Thus, school surroundings are receiving increased attention because children and adolescents frequent food outlets on their way to and back from school, which may impact their food choices [17,18,19]. Indeed, adolescents leave school boundaries during breaks to buy foods and drinks [20].

Assessments of the food environment surrounding schools are common in the literature. However, most studies have been conducted in Anglo-Saxon settings such as the US, the UK, New Zealand, or Australia [21,22,23,24]. A study in the US found that adolescents obtained more than 90% of their total calories from outside the school setting [23]. Another study conducted in New Zealand showed that more than 60% of urban schools had an unhealthy food outlet within walking distance [22]. A recent UK longitudinal study found a positive association between the count of food retailers and adolescents’ weight status [24]. Although effect sizes were small, these findings taken together highlight the potential impact of the food environment surrounding children’s schools on children’s food choices, diet quality, and body weight.

As a result, zoning policies to restrict unhealthy food retailing are being proposed and implemented [25]. Indeed, the New London Plan includes a new policy (Policy E9) stating that “hot food takeaways should not be permitted to be located within 400 m walking distance of an existing or proposed primary or secondary school” [26]. Similar to London, where 38% of children are overweight or obese, one in four children living in Madrid or Barcelona have excess body weight [5,26]. Adopting a Health in All Policies approach, Franco et al. identified a series of multisector policy changes that may help tackle childhood obesity in Spain [27]. Yet, the Spanish Strategy for Nutrition, Physical Activity, and the Prevention of Obesity (NAOS strategy) appeals to parents’ responsibility and voluntary self-regulation of the food industry [28]. In addition, previous research has shown that, in regard to the nutritional quality of the products sold in vending machines within schools, compliance with NAOS recommendations is low [29].

Moreover, area-level socioeconomic status (SES) has been linked to spatial patterns of retailers’ locations. Across the US, according to Zenk et al., schools located in more disadvantaged areas had 32% more fast food retailers than those located in more advantaged areas [30]. In New Zealand, unhealthy food access (within walking distance) was also greater from urban schools located in more deprived areas than from schools located in the leastdeprived areas [22]. Yet, studies conducted across European contexts have shown mixed results. For example, Timmermans et al. found that unhealthy options were the default around schools in the Netherlands [20]. Yet, they found few differences by area-SES level [20]. To date, no research assessing the environment surrounding children and adolescents (e.g., the food environment around schools) has been conducted in Spain. However, retail food environments have been shown to vary widely across geographical settings [31,32,33].

To fill this gap, our aim was to assess the spatial access to an unhealthy food environment around schools across the city of Madrid (Spain) and to examine its association with neighborhood-level socioeconomic status.

## 2. Materials and Methods

### 2.1. Study Design and Sample

This study was nested within the European-funded project “Heart Healthy Hoods”, which assesses the association between the urban environment (including the food environment) and cardiovascular outcomes [34]. We did not require any institutional review board for this study because no human participants were involved.

Our study area comprised the entire city of Madrid, which was administratively divided into 21 Districts, 128 neighborhoods, and 2443 census tracts in 2017. For reference, census tracts are the smallest geographic units for which population data are released in Spain. Their average population size was 1323 residents in 2017 [35]. The city of Madrid covers a total area of 60,577 km², has a population density of 525,444 hab./km², and 14.2% of residents are of age between 3 and 18 years.

Schools were our spatial unit of analysis. We included all schools in the city, limiting the age range of children attending school to 3–18 years. We classified schools into (1) preschools, including children 3 to 6 years old; (2) primary schools, from the ages of 6 to 11 years (which may also offer preschool); and (3) secondary schools, including adolescents from 12 to 18 years, which may also offer preschool and primary education. We included public and private (including both independent private and partially publicly funded private) schools. The latter (“concerted” schools) are private schools receiving regional government support to provide education services, which follow the same rules as schools in the public system.

Data were obtained from the Department of Education of Madrid’s Government (for 2017). Each school was mapped using the geographic x and y coordinates and entered into a Geographic Information System, using ArcGIS software 10.3. We then overlaid school points to census tract boundaries using a spatial join. The latter allowed us to identify the fitting census tract for each school.

### 2.2. Neighborhood-Level Socioeconomic Status

Our main exposure of interest was neighborhood-level socioeconomic status (NSES). Consistent with previous studies [36,37], we measured NSES using a composite index constructed at census tract level. This NSES index included seven indicators: (1) low education (% people above 25 years with primary studies or below); (2) high education (% people above 25 years with college or university studies); (3) part-time work (% workers in part-time jobs); (4) temporary work (% people aged 16 years or over in temporary jobs); (5) manual work (% of people aged 16 years or over working in manual or unqualified jobs with respect to the total employed population aged 16 or over); (6) unemployment (% of residents aged 16 years or over registered as unemployed among residents aged 16–64 years); and (7) average housing prices (€/m^2^). These indicators relate to the four domains (education, occupation, living conditions, and wealth) suggested for the study of the effect of structural policies on health inequalities in Spain [38].

Further details on the development and use of this SES index have been previously published [36,37]. In brief, we collected data from a combination of administrative databases (the municipal census, social security, and employment services registries) and from a real estate company (IDEALISTA). All administrative data were freely available for the year 2017, except for the average housing prices, which were available for 2016.

For each indicator, we centered to the mean and divided it by the standard deviation (of all census tracts in Madrid) to obtain a z-score of each indicator. We then averaged z-scores of each indicator, resulting in a z-score for each domain (education, wealth, occupation, and living conditions); and finally, we calculated the composite index of NSES by summing the z-score of each of the four domains. We operationalized the NSES measure as a categorical variable using quintiles based on the SES index score distribution across census tracts: Low (Q1), middle low (Q2), middle (Q3, reference category), middle high (Q4), and high (Q5).

### 2.3. Food Environment Assessment

Our main outcome was the spatial access to unhealthy retailers around schools in terms of (1) availability (counts) of retailers selling unhealthy foods and beverages within 400 m; and (2) distance to the nearest unhealthy retailer (in m).

We defined as unhealthy foods both energy-dense, nutrient-poor food products (e.g., potato chips, chocolate) and sugar-sweetened beverages (beverages containing caloric sweeteners). Although there is no universally accepted classification (or definition) of unhealthy food retailers, ours is consistent with previous studies examining food stores featuring unhealthy food [20,39].

Food retail data for 2017 came from a secondary database of the Department of Statistics of Madrid City Council (*Censo de Locales y Actividades*) covering all licensed premises citywide. This information is collected for statistical purposes, licensing, and inspections. It is freely available, yearly updated, and collects name, location, and type for each premise. Retailer types are coded following the statistical classification of economic activities in the European Community (NACE) [40]. For example, according to the NACE codes for food retail, any premise including retail sale (not for consumption on the premises) of bread, cakes, flour confectionery, and sugar confectionery in specialized stores is coded as a bakery.

Building on previous research using this dataset [39], we identified 6530 outlets within the scope of the study (selling any unhealthy food products): nonspecialized retailers (e.g., supermarkets or convenience stores), specialized retailers (e.g., bakeries), and food services (e.g., fast-food restaurants or take-away-only restaurants). We excluded full-service restaurants and outlets within closed facilities (e.g., airports) as children are unlikely to use them. Table S1 shows the food retailers considered for each category.

We defined unhealthy food availability by counting all unhealthy outlets within a 400 m Euclidian buffer surrounding each school, which reflects a 5 min walking distance. We also determined accessibility by calculating Euclidean distance measures (in meters) from school locations and the nearest “unhealthy” outlet, by performing a Near tool of ArcGIS (ArcGIS 10.3, ESRI). There is empirical evidence that suggests 400 m (or about a 5 min walk) [41] is the distance students are most likely to walk (on foot) during a short break. This small distance has previously been used in food environment research [20,42,43].

### 2.4. Population Density

Consistent with previous research [44,45] showing that certain types of food stores (e.g., fast-foods) are often co-located in areas of high residential density, we used population density to control for urban factors in our models. We specified population density, at the census tract level, by dividing the number of residents over the land area of the census tract. Population data came from the 2017 municipal registry. We downloaded the information for the administrative boundaries (including land area of the census tracts) from the Spanish National Mapping Agency.

### 2.5. Statistical Analysis

We used the Kruskal–Wallis test to compare medians for school characteristics (school type, funding, NSES, and population density) according to both count and distance measures. We then assessed the association between NSES and both dependent variables (counts of unhealthy outlets and distance to the nearest unhealthy outlet).

To model the counts of unhealthy food outlets, we used a multilevel negative binomial regression model. We used a negative binomial regression (instead of a Poisson regression) because the data were over-dispersed and zero-inflated [46]. To model the distance to the nearest unhealthy outlet, we log-transformed the variable and fit a multilevel linear mixed model. We transformed the distance variable because of its extreme right skewness, and thus, non-normal distribution. For all models, the unit of analysis was the school. To assess the need for random intercepts at different levels, we fit empty models and progressively added random intercepts for census tract (*n* = 2443), neighborhood (*n* = 128), and district (*n* = 21), and compared models using the AIC (Akaike Information Criterion). The final models had 4 levels, with random intercepts for the three administrative units. We then fit unadjusted models, with NSES quintiles as the main independent variable, and adjusted models with population density as a covariate.

In addition, we ran sensitivity analyses for both outcomes (counts of and distance to unhealthy retailers) without considering supermarkets. Supermarkets are difficult to categorize as unhealthy retailers because they offer a wide variety of both healthy (e.g., fresh fruits) and unhealthy food products (such as chips, unhealthy ready meals, sugar-sweetened beverages, etc.) [47,48]. We conducted all analyses using STATA/SE 15 (StataCorp., College Station, TX, USA).

## 3. Results

We identified 1321 schools. About a third of them were private (37%) or concerted (27.5%), while 35.4% were public schools. Preschools represented 45.6% of all schools, whereas 22.2% were primary schools and 32.2% were secondary schools. For reference, roughly 36% of all preschoolers and 37% of children attended public preschools and primary schools, respectively, during the academic year 2017–2018. Approximately 50% did so in secondary schools for the same year (data not shown).

### 3.1. Counts of Unhealthy Food Outlets and Neighborhood-level SES

We found that 94.9% of the schools had unhealthy outlets within 400 m. As shown in Table 1, there was a clear gradient in terms of NSES and the median number of unhealthy outlets per school. The median number of available unhealthy food outlets around the schools located in low-NSES areas was greater (median = 24) than those located in high-NSES ones (median = 8; *p* = 0.0001). We also found differences in terms of the type of school. Concerted schools had higher counts of unhealthy food outlets (median = 23) relative to those publicly-funded schools (median = 16; *p* = 0.0001).

**Figure 1:**
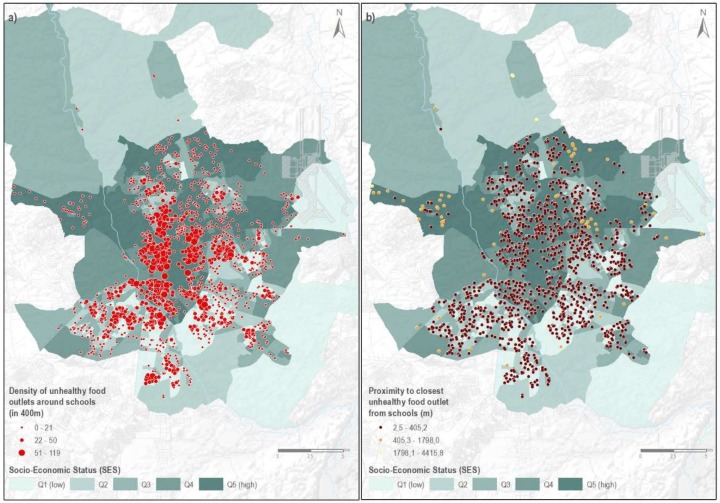
Schools in the city of Madrid (2017): (**a**) by availability of unhealthy outlets around schools; (**b**) by schools’ proximity to the closest unhealthy food source.

Figure 1a shows the spatial distribution of schools citywide, according to their availability of unhealthy retailers within a 400 m buffer. We found an east-west divide, with schools located in eastern parts of the city showing lower counts of retailers than schools located in the western part of the city. Schools located in suburban areas of the city also appeared to perform better (e.g., less unhealthy food sources around them) than those located in the city center.

**Figure 2:**
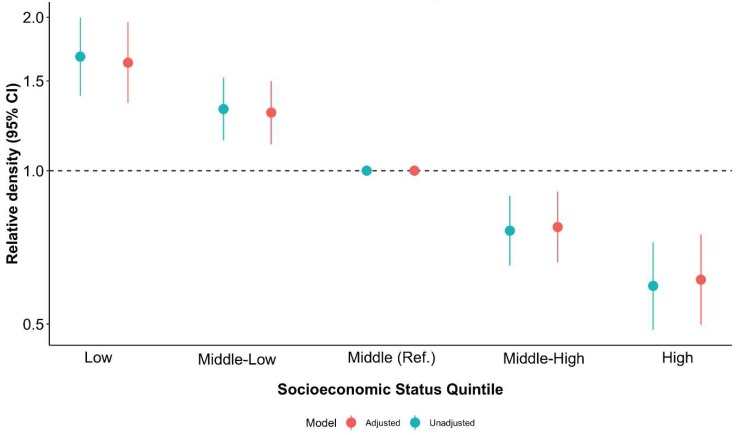
Multilevel negative binomial regression modelling neighborhood-level socioeconomic status with counts of unhealthy outlets around schools in Madrid (2017) before and after adjusting for population density.

Figure 2 shows the results from the binomial regression model assessing the socioeconomic correlates of unhealthy retailers’ availability. As such, it depicts the results of the models on NSES, both adjusted (in red) and unadjusted (in blue) for population density. Figure 2 illustrates the relative availability: that is, the ratio of the count of food retailers (within 400 m) in each NSES category over the count of food retailers in the middle-NSES quintile.

Schools located in the most disadvantaged census tracts showed, on average, 67% more counts of unhealthy retailers (IRR (Incidence Rate Ratio) = 1.67; 95% CI (Confidence Interval) = 1.40, 1.99) than those located in middle-NSES areas (reference category). In contrast, schools located in more advantaged census tracts showed, on average, 41% fewer counts of unhealthy retailers (IRR = 0.59; 95% CI = 0.48, 0.72) than those in middle-NSES areas. After adjusting for population density, the effect of neighborhood-level socioeconomic status remained inversely associated with the counts of unhealthy retailers around schools (shown in red). For instance, schools located in low-NSES areas (two lowest Qs) were associated with greater counts, on average, of 29% (middle-low, IRR = 1.29; 95% CI = 1.12, 1.50) and 62% (low, IRR = 1.62; 95% CI = 1.35, 1.95) of retailers than those located in middle-NSES areas. Thus, population density did not change the NSES gradient observed in the unadjusted analyses. Table S2 shows further details of these results. In our sensitivity analysis, results remained the same when removing supermarkets from the counts of unhealthy retailers (see Table S3).

### 3.2. Distance to the Nearest Unhealthy Outlet and Neighborhood-Level SES

As shown above, in Table 1, the median distance from schools located in more disadvantaged areas was smaller (77.09 m), relative to those located in more advantaged ones (145.92 m; *p* = 0.0001). On average, any school in Madrid was located within 88 m of an unhealthy retailer (median: 88.3 m; range: 2.53–4415 m). Figure 1b shows the spatial distribution of schools in the city, according to their proximity to the closest unhealthy food outlet. As shown in Figure 1b, we did not observe any clear spatial pattern in terms of distance to unhealthy retailers across the city.

**Figure 3:**
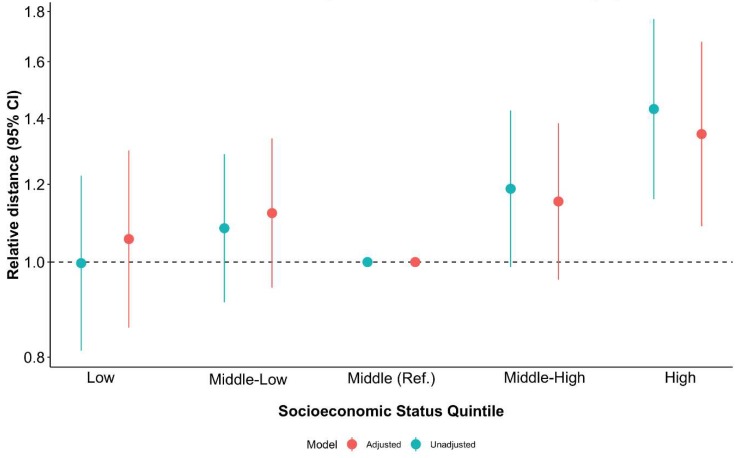
Multilevel linear regression modeling neighborhood-level socioeconomic status with distance to unhealthy retailers (in the log scale) from schools across Madrid (2017) before and after adjusting for population density.

Figure 3 presents the results of models of log (distance) on NSES (adjusted and unadjusted for population density). Therefore, it is a measure of relative distance: that is, the ratio of the distance to the closest unhealthy retailer in each NSES category over the distance to the closest unhealthy retailer in the middle-NSES category.

Schools located in more advantaged areas showed greater distances to the nearest unhealthy food retailer than those schools located in middle-NSES areas (unadjusted model shown in blue). Indeed, a unit change in the NSES represents an expected increase in the distance to the nearest unhealthy outlet of 41.9%. As we log-transformed the distance variable, the beta coefficients represent percentage changes.

The association remained nearly identical in direction and magnitude after controlling for population density (adjusted model shown in red). Having a school located in more disadvantaged areas (two lowest Qs) was negatively associated with the distance to unhealthy outlets, but results showed weak evidence (middle-low, β= 0.11; 95% CI = −0.06, 0.29; low, β = 0.05; 95% CI = −0.15, 0.26). Table S4 shows further details of these results. In our sensitivity analysis and as with counts of unhealthy food outlets, results remained the same after removing supermarkets from the distance metrics (see Table S5).

## 4. Discussion

Our findings suggest that schools located in socioeconomic disadvantaged areas have a higher availability of unhealthy outlets in their immediate food environment than schools located in socioeconomic advantaged areas. Indeed, schools located in more disadvantaged areas showed, on average, 62.0% more counts of unhealthy retailers than those located in middle-NSES areas. When we excluded supermarkets, we still observed that schools located in more disadvantaged areas showed, on average, 69.0% more counts than those located in middle-NSES areas. Furthermore, and although not statistically significant, schools located in disadvantaged areas were closer to “unhealthy” outlets than school located in more advantaged areas.

By building on previous studies [3,4], these findings contribute to the literature by assessing the food environment around schools across a Southern European context, where children and adolescents present particularly high prevalence of overweight and obesity. To the best of our knowledge, this is the first study to examine the retail food environment surrounding schools across an entire Southern European city like Madrid. According to a recent systematic review by Bivoltsis et al. [49], there is no current consensus on the use of different exposure measures for food environment. Therefore, following their recommendations, we used a multimethod approach and decided to measure the effect of two dimensions of spatial access, availability and accessibility. These two measures of spatial access are the environmental correlates consistently found to be associated with children’ and adolescents’ dietary intake [50,51,52,53]. Further, they are interconnected and complementary.

Our measure of availability (counts) produced more important associations than our measure of accessibility (distance). These results are in line with the ones shown in the previous review [49], documenting effect sizes from accessibility measures to be smaller than effects from availability measures. Prior research has also shown different associations between neighborhood-level socioeconomic status and food access according to the accessibility dimension measured [54]. These two measures do also provide different insights from a policy perspective. Availability metrics (either raw counts or densities) are more relevant for implementing food policies, such as zoning policies. Across urban, dense, areas the number of unhealthy outlets (i.e., the concentration of these outlets) may have a greater influence on diet than the distance required to get to the closest unhealthy outlet. 

In this study, we assessed multiple retailer types (supermarkets, convenience stores, bakeries, fast-food outlets, takeaways, etc.) to capture the wider experience of environmental features promoting unhealthier dietary intakes. Previous research has focused on whether specific food retailers (e.g., fast foods) were associated with youth excess weight risk [21]. However, children and adolescents interact with multiple types of retailers simultaneously. By including all sources of retailers offering unhealthy food products (including supermarkets), we help to better identify sources for purchasing unhealthy food and beverages options within schools’ surroundings [55].

While supermarkets are often used as a proxy for “healthy” food options, they also stock a wide variety of unhealthy items. Indeed, previous studies (conducted in the US) have documented this wide availability of unhealthy options inside supermarkets, which leads to an increased intake of these energy-dense, nutrient-poor foods and beverages [56,57]. Moreover, Howard Wilsher et al. showed an association between supermarket sales of unhealthy foods and the prevalence of excess weight among children in the UK [58]. In other countries, as diverse as Brazil [59] and Switzerland [60], researchers have also documented how supermarkets are rapidly becoming the main source of ultra-processed foods. We found similar results when omitting supermarkets (see Tables S3 and S5).

Our findings support the “deprivation amplification” hypothesis, where socially disadvantaged individuals experience a further contextual disadvantage regarding their access to health-promoting resources due to their place of residence [61]. Indeed, our findings concur with previous studies [22,30,62,63] showing that unhealthy food options are the default around schools in urban settings, with this being more the case for schools located in more disadvantaged areas. For example, Soltero et al. examined the food environment around schools in three Mexican cities and showed that the food environment was saturated with unhealthy food stores (range 2–273 retailers) [63]. Regarding the socioeconomic gradient, the Soltero et al. study also found differences in all three cities by education and poverty levels. In Madrid, a recent study showed that youth living in more disadvantaged areas had greater odds of being obese compared with those living in more advantaged areas [64]. Yet, to date, there is no research for Southern European cities like Madrid, assessing whether these social disparities in obesity are associated with the food environment surrounding children and adolescents.

Moreover, prior studies have documented how supermarkets are becoming more prevalent in Spain, whereas traditional specialized food stores (e.g., fruit and vegetable stores) are disappearing [39,65]. These changes in the retail food environment might also impact dietary intake, and further, diet-related health outcomes. In Madrid, previous studies have documented that traditional stores provide urban residents with a greater ratio of fresh and/or healthy food, as compared with supermarkets [34,39]. In addition, Thornton et al. documented the ubiquity of unhealthy foods across supermarkets in Melbourne [66]. Furthermore, a recent study in Cape Town showed that socioeconomic status may play a role on the food available in supermarkets. This study showed that supermarkets in low-SES areas carried fewer healthy foods than supermarkets in higher-SES areas [67]. Thus, the potential implications that the proliferation of supermarkets may have on nutrition and health deserves attention.

The specific mechanisms by which the retail food environment influences children and adolescent food choices are not fully understood and may include different physical, sociocultural, economic, and political factors [9,10,11]. Physical factors (e.g., access to fast-food outlets) have been suggested to be positively associated with dietary behaviors or BMI (Body Mass Index) [68,69,70]. For instance, Cutumisu et al. found higher access to fast-food outlets around schools to be positively associated with higher intake of junk food [68]. In Finnland, Virtanen et al. found proximity to unhealthy retailers to be associated with eating snacks obtained from outside the school [69]. Baek et al. showed an increase (by 0.004 units) of children’ BMI z-scores per each additional convenience store available within walking distance to public schools in California [70].

Our study findings underscore the need for examining childhood obesity from a social justice perspective. This need urges researchers, urban planners, and decision-makers to work together towards tackling the childhood obesity epidemic using a multiple systems approach. While the evidence is still inconsistent, several countries have introduced regulatory policies to restrict unhealthy food retailing [71,72,73]. Across the city of London, new hot food takeaways should not be permitted within 400 m walking distance of an existing school [26]. However, our findings showed that the median number of unhealthy retailers varied across schools located in high-, middle-, and low-SES neighborhoods (8, 16, and 24, respectively; Table 1). Thus, restricting only the location of new hot food takeaways may have little effect for those living in the most disadvantaged areas, compared with the effect for those living in the least disadvantaged areas. Developing food policies which promote healthier food environments, as well as considering wider health inequalities, may be more appropriate from a health-equity lens than restricting the location of new fast-food outlets. As shown in our results, and as also highlighted by Green et al., disadvantaged areas already have a level of saturation whereby restricting further outlets would likely have little impact [24]. This brings a further disadvantaged scenario for children and adolescents living in these areas. Thus, planning and zoning policies should include the existing retailers.

Our study has some limitations. We assumed that children attend schools within their neighborhood. However, this may or may not be the case. No conclusions about individuals can be drawn from this study. Another limitation is that we focused on unhealthy outlets within school surroundings, which is not fully representative of the school food environment, as vending machines and cafeterias within school boundaries are missing. While our unhealthy food environment measure is comprehensive, we assumed that food retailers are either healthy or unhealthy, which may be simplistic. Further, it is also important to account for other aspects of the retail food environment (such as the prices of food products or the opening hours of the retailers). Using administrative units to measure both SES and population density (at the census tracts in our study) also means results are subject to the modifiable areal unit problem [35]. Finally, it must be kept in mind that individuals residing in these areas would have additional exposure to fast-food restaurants beyond these areas. Taken together, these limitations may have underestimated our results. However, despite these limitations, our study has several strengths. This study is the first to assess the unhealthy food environment around schools in a Southern-European setting, characterized by the density of food retailers [31]. We have also used a validated neighborhood-level SES measure using an integrated composite index [35]. Our unhealthy food access measures include multiple types of food store types, yielding a more comprehensive picture of the food environment.

Future studies should try to extend our results by including individual-level data (e.g., dietary intake) to better understand how the food environment surrounding schools relates to actual food-purchasing practices and diet quality. Further, a wider range of spatial access dimensions should be assessed (e.g., affordability) to increase our understanding of how these spatial metrics are associated with food-purchasing practices.

## 5. Conclusions

Our study shows that schools located in more disadvantaged areas have a higher exposure to unhealthy outlets in their immediate food environment than schools located in more advantaged areas. Restricting policies (e.g., banning fast-food around schools) are warranted to protect children and adolescent from the harmful effects of unhealthy foods and beverages. Yet, it is important that public policy interventions consider the influence that socioeconomic inequalities have on the retail food environment.

## Figures and Tables

**Table 1:** Counts (within 400 m) of and distance (m) to unhealthy retailers across schools (*n =* 1321) citywide (Madrid, 2017).

Characteristics	Availability		Distance	
	Median (IQR) ^1^	*p*-Value ^2^	Median (IQR) ^1^	*p*-Value ^2^
**Type of school**		**0.0037**		**0.0011**
Preschools	16 (8–31)		82 (48.10–151.85)	
Primary schools	18 (9–36)		90.07 (57.93–141.01)	
Secondary schools	17 (8–35)		100.80 (55.21–171.13)	
**Funding**		**0.0001**		**0.0001**
Public	16 (9–29)		100.78 (62.40–153.68)	
Private	14 (5–31)		90.41 (47.61–188.85)	
Concerted	23 (12–44)		74.16 (48.22–126.77)	
**Neighborhood-level SES**		**0.0001**		**0.0001**
Low-SES	24 (13–37)		77.09 (46.50–129.26)	
Middle-low	18 (13–30)		81.35 (54.65–128.57)	
Middle	16 (8–36)		77.52 (43.59–127.63)	
Middle-high	19 (7–43)		92.01 (52.83–162.77)	
High-SES	8 (3–16)		145.92 (77.48–254.71)	
**Population density** (10^3^ residents/km^2^)		**0.0001**		**0.0001**
Low	22 (13–39)		71.52 (44.85–125.07]	
Medium	21 (10–39)		83.99 (51.85–139.69]	
High	10 (4–21)		123.26 (69.24–193.26]	

^1^ IQR=interquartile range, ^2^ *p*-values correspond to Kruskal–Wallis test. SES: socioeconomic status.

## Supplementary Materials

The following are available online at https://www.mdpi.com/2072-6643/11/7/1511/s1, Table S1: Unhealthy food retailers (by type) found within 400 m of schools (*n* = 6530) citywide (Madrid, 2017), Table S2: Association between neighborhood-level socioeconomic status and counts of unhealthy retailers, using multilevel negative binomial regression, Table S3: Sensitivity analysis: Association between neighborhood-level socioeconomic status and counts of unhealthy retailers (without including supermarkets), using multilevel negative binomial regression, Table S4: Association between neighborhood-level socioeconomic status and distance to the closest unhealthy retailer (logarithm), using multilevel linear regression. Table S5: Sensitivity analysis: Association between neighborhood-level socioeconomic status and distance (logarithm) to the closest unhealthy retailer (without including supermarkets), using multilevel linear regression.
