# Supplementary material for: Socioeconomic Inequalities in the Retail Food Environment around Schools in a Southern European Context
Source: Nutrients. 2019 Jul 3;11(7):1511. doi: 10.3390/nu11071511 (PMC6683257; doi:10.3390/nu11071511)
Supplement: Supplementary file 1 [file nutrients-11-01511-s001.pdf]

Table S1. Unhealthy food retailers (by type) found within 400-m of schools (n= 6530) city-wide (Madrid, 2017).

	n	%
Supermarkets	1005	15.39
Grocery stores	2176	33.32
Convenience stores	54	0.83
Bakeries (for immediate consumption)	1367	20.93
Candy and confectionary stores	97	1.49
Fast-foods (e.g., McDonalds)	805	12.33
Coffee shops (e.g., selling packaged foods, hot chocolate, etc)	184	2.82
Ice-cream shops	124	1.90
Kiosks	22	0.34

Table S2. Association between neighborhood-level socioeconomic status and counts of unhealthy retailers, using multilevel negative binomial regression.

	**Undjusted models** [1] (n = 1321 schools)		**Adjusted model** [2] (n = 1321 schools)	
	IRR [3]	95% CI	IRR [3]	95% CI
**Neighborhood-level SES**				
High	0.59 ***	[0.48, 0.72]	0.61 ***	[0.49, 0.74]
Middle-High	0.76 ***	[0.65, 0.89]	0.77 ***	[0.66, 0.90]
Middle	-ref-		-ref-	
Middle-Low	1.32 ***	[1.14, 1.52]	1.29 ***	[1.12, 1.50]
Low	1.67 ***	[1.40, 1.99]	1.62 ***	[1.35, 1.95]
**Population density** (10^3 residents/km^2)	0.98 ***	[0.97, 0.99]	0.99	[0.98, 1.00]

[1] Estimates obtained from separate unadjusted negative binomial models, [2] All estimates are mutually adjusted for all variables listed, [3]IRR=incidence rate ratio; 95% CI = 95% confidence interval. * $p < 0.05$, ** $p < 0.01$ *** $p < 0.001$

Table S3. Sensitivity analysis: Association between neighborhood-level socioeconomic status and counts of unhealthy retailers (without including supermarkets), using multilevel negative binomial regression.

	**Undjusted model** [1] (n = 1321 schools)		**Adjusted model** [2] (n = 1321 schools)	
	IRR [3]	95% CI	IRR [3]	95% CI
**Neighborhood-level SES**				
High	0.57 ***	[1.44, 2.07]	0.59 ***	[0.47, 0.72]
Middle-High	0.74 ***	[0.63, 0.87]	0.75 **	[0.64, 0.88]
Middle	-ref-		-ref-	
Middle-Low	1.35 ***	[1.17, 1.56]	1.34 ***	[1.15, 1.55]
Low	1.73 ***	[1.44, 2.07]	1.69 ***	[1.40, 2.04]
**Population density** (10^3 residents/km^2)	0.98 ***	[0.97, 0.99]	0.99	[0.98, 1.00]

[1] Estimates obtained from separate unadjusted negative binomial models, [2] All estimates are mutually adjusted for all variables listed, [3]IRR=incidence rate ratio; 95% CI = 95% confidence Interval, * $p < 0.05$, ** $p < 0.01$ *** $p < 0.001$.

Table S4. Association between neighborhood-level socioeconomic status and distance to the closest unhealthy retailer (logarithm), using multilevel linear regression.

	Undjusted models [1] (*n* =1321 schools)		Adjusted model [2] (*n* = 1321 schools)	
	**B** [3]	95% CI	β [3]	95% CI
**Neighborhood-level SES**				
High	0.35 ***	[0.14, 0.57]	0.30 **	[0.08, 0.51]
Middle-High	0.17	[-0.01, 0.35]	0.14	[-0.04, 0.32]
Middle	-ref-		-ref-	
Middle-Low	0.07	[-0.09, 0.25]	0.11	[-0.06, 0.29]
Low	- 0.01	[-0.20, 0.20]	0.05	[-0.15, 0.26]
**Population density** (10^3 residents/km^2)	0.02 ***	[0.01, 0.02]	0.01 *	[0.00, 0.02]

[1] Estimates obtained from separate unadjusted negative binomial models, [2] All estimates are mutually adjusted for all variables listed, [3] β = Coefficient estimates represent percentage changes due to the natural logarithm transformation applied to distance, the dependent variable; 95% CI = 95% confidence Interval, * $p < 0.05$, ** $p < 0.01$ *** $p < 0.001$.

Table S5. Sensitivity analysis: Association between neighborhood-level socioeconomic status and distance (logarithm) to the closest unhealthy retailer (without including supermarkets), using multilevel linear regression.

	Undjusted models [1] (*n* = 1321 schools)		Adjusted model [2] (*n* = 1321 schools)	
	β [3]	95% CI	β [3]	95% CI
**Neighborhood-level SES**				
High	0.43 ***	[0.22, 0.64]	0.37 **	[0.15, 0.58]
Middle-High	0.19*	[0.01, 0.37]	0.16	[-0.02, 0.34]
Middle	-ref-		-ref-	
Middle-Low	- 0.01	[-0.01, 0.55]	0.02	[-0.15, 0.19]
Low	- 0.11	[-0.32, 0.08]	-0.06	[-0.26, 0.14]
**Population density** (10^3 residents/km^2)	0.02 ***	[0.01, 0.03]	0.01 *	[0.00, 0.02]

[1] Estimates obtained from separate unadjusted negative binomial models, [2] All estimates are mutually adjusted for all variables listed, [3] β = Coefficient estimates represent percentage changes due to the natural logarithm transformation applied to distance, the dependent variable; 95% CI = 95% confidence Interval, * $p < 0.05$, ** $p < 0.01$ *** $p < 0.001$.
